# Metachronous EBV-positive classical Hodgkin lymphoma following EBV-positive diffuse large B-cell lymphoma: a case report and literature review

**DOI:** 10.3389/fonc.2026.1867889

**Published:** 2026-08-20

**Authors:** Jiayu Li, Dajie Zhou, Jing Wang, Guohua Yu

**Affiliations:** 1 Department of Pathology, The Affiliated Yantai Yuhuangding Hospital of Qingdao University, Yantai, Shandong, China; 2 Department of Clinical Laboratory, The Affiliated Yantai Yuhuangding Hospital of Qingdao University, Yantai, Shandong, China

**Keywords:** classical Hodgkin lymphoma, composite lymphoma, diffuse large B-cell lymphoma, EBV, metachronous

## Abstract

Epstein-Barr virus (EBV) is closely associated with the development of various malignancies, particularly in lymphomas. Diffuse large B-cell lymphoma (DLBCL), as the most common subtype of non-Hodgkin lymphoma, and classical Hodgkin lymphoma (CHL) both originate from B cells, yet they exhibit significant differences in pathological diagnosis and clinical management. While composite lymphoma (CL) with sequential or simultaneous DLBCL and CHL has been reported, cases where both components are EBV-positive remain rare. Here we report a rare case of composite lymphoma with EBV-positive DLBCL followed by EBV-positive CHL. The patient was a 35-year-old male diagnosed with EBV-positive DLBCL in 2019. After one cycle of CHOP regimen followed by seven cycles of DA-EPOCH-R regimen, he achieved clinical complete remission. Sixty-nine months later, the patient was readmitted due to multiple lymphadenopathy, and the pathological diagnosis was EBV-positive CHL (mixed cellularity type). After two cycles of ABVD chemotherapy, he achieved partial remission, but later showed disease progression and is still under treatment. We discuss this rare case in conjunction with literature review, aiming to provide reference for the diagnosis and treatment of similar cases.

## 1 Introduction

Composite lymphoma (CL) refers to the simultaneous or sequential occurrence of two or more different types of lymphoma in the same or different sites of the same patient, with an annual incidence accounting for only 1% to 4.7% of all new lymphoma cases (1, 2). Among them, CL composed of classical Hodgkin lymphoma and non-Hodgkin lymphoma is even rarer, with a much lower incidence than other combination forms (3). EBV infection is closely associated with the development of various lymphomas and may play an important role in the pathogenesis of CL (4). Synchronous CL refers to the simultaneous occurrence of two lymphomas in the same or different sites, while metachronous CL refers to the sequential appearance of the two in the same or different sites. However, cases of combined EBV-positive DLBCL and CHL, with both components being EBV-positive, are extremely rare, and their clinicopathological features and treatment strategies remain unclear. This article reports a case of metachronous EBV-positive DLBCL followed by EBV-positive CHL, and through a review of the literature, aims to enhance the understanding of this rare disease.

## 2 Case report

**Figure 1:**
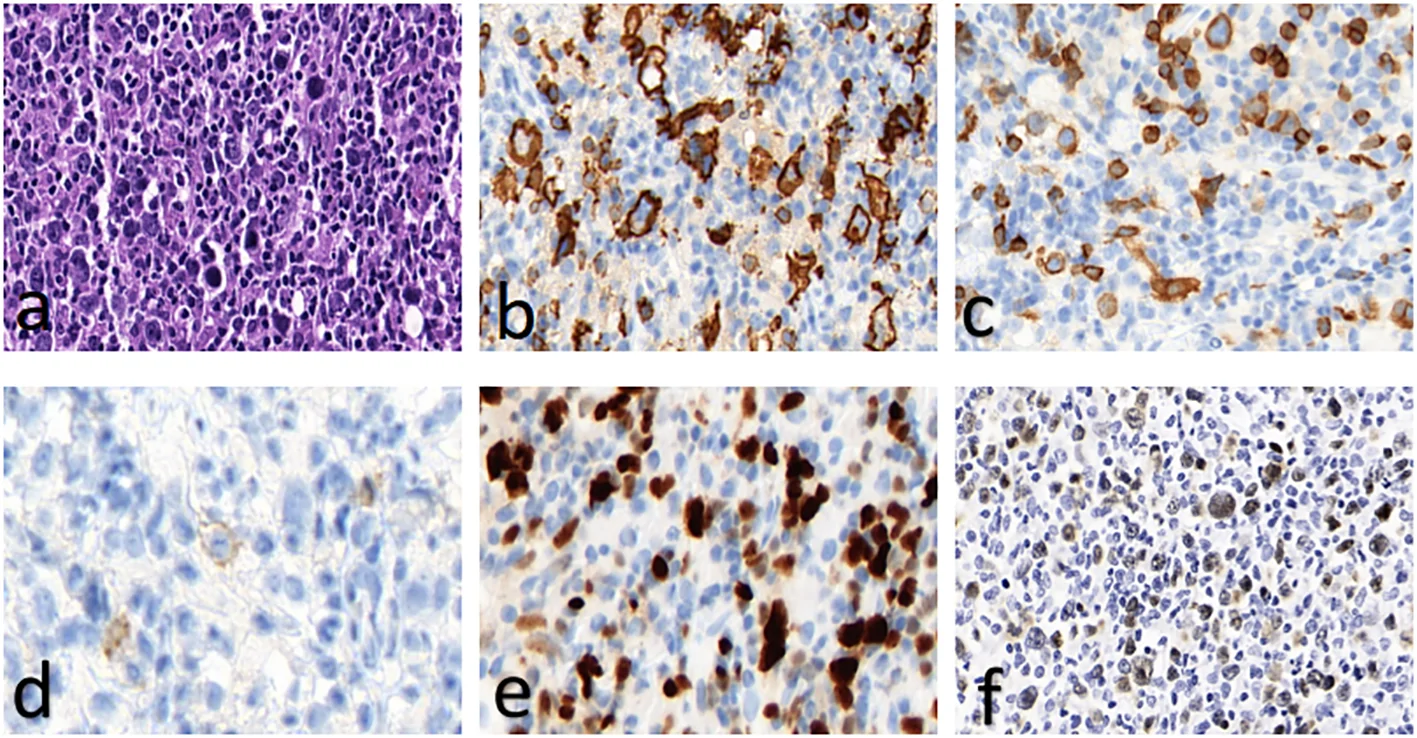
Pathological examination. a Microscopic findings (hematoxylin-eosin (HE) staining) show destruction of normal lymph node architecture with diffuse proliferation of abnormally large tumor cells exhibiting swollen nuclei, frequent pathological mitotic figures, abundant basophilic cytoplasm, and marked cellular atypia, scattered within a reactive background. The tumor cells were positive for CD20 **(B)**, CD79a **(C)**, CD30 **(D)**, and Ki-67 **(E, F)** ISH analysis demonstrated positivity for Epstein-Barr virus-encoded small RNA in approximately 80% of tumor cell nuclei. Original magnification: **(A)** ×55.5; **(B)** ×80.0; **(C)** ×80.0; **(D)** ×100.0; **(E)** ×63.0; **(F)** ×40.0.

The patient, a 35-year-old male, was admitted to the Otorhinolaryngology Department in October 2019 due to a neck mass that had been present for one month and a groin mass that had been noted for 10 days. Biopsies of the nasal cavity mass and neck mass were performed, and the pathological morphology and immunohistochemical findings of the two sites were essentially consistent. Microscopic examination revealed a lymphoproliferative disorder with effacement of normal nodal architecture. There was diffuse proliferation of abnormally large tumor cells exhibiting swollen nuclei, frequent pathological mitotic figures, abundant basophilic cytoplasm, and marked cellular atypia, scattered within a reactive background (**Figure 1A**). Immunohistochemical staining showed that the tumor cells were positive for CD20 (**Figure 1B**), CD79a (**Figure 1C**), CD30 (**Figure 1D**), MUM1, and BCL-2, with a Ki-67 positivity rate of 80% in tumor cells (**Figure 1E**). CD21 revealed a few residual FDC networks. The tumor cells were negative for CK, EMA, CD10, BCL-6, ALK, CD3, CD4, and CD5. EBV-EBER *in situ* hybridization (ISH) showed that approximately 80% of tumor cell nuclei were positive (**Figure 1F**). Based on the morphological and immunohistochemical findings, the lesion was diagnosed as EBV-positive DLBCL. After completion of pathological classification, full immunohistochemical panel, and EBER *in situ* hybridization, the remaining tissue from the paraffin-embedded specimen was insufficient for IGH gene rearrangement clonality assay. The patient’s hepatitis B virus serological tests showed: HBsAg >250 IU/mL, HBeAg 1023.720 S/CO, Anti-HBc 8.65 S/CO, indicating hepatitis B virus infection with active viral replication. At initial diagnosis, the patient had a high tumor burden, with multiple masses in the neck and inguinal region accompanied by increased bone metabolism, and the Ann Arbor stage was IVA. The patient could perform normal activities (ECOG performance status 0). Prognostic assessment: IPI score 3 (intermediate-high risk), aaIPI score 2 (intermediate-high risk). To reduce the risk of serious complications such as tumor lysis syndrome, the clinical decision was to administer only the Cyclophosphamide, Hydroxydaunomycin, Oncovin, Prednisone (CHOP) regimen in the first cycle for debulking. After debulking, because the patient was young and the EBV-positive subtype carries a relatively poorer prognosis, a sequential 7 cycles of the Rituximab, Dose-adjusted Etoposide, Prednisone, Oncovin, Cyclophosphamide, Hydroxydaunomycin (DA-EPOCH-R) regimen was given to achieve deep remission. After treatment, clinical complete remission was achieved. Subsequently, the patient was subsequently lost to regular follow-up.

After 69 months, the patient was admitted to the Department of Hematology of our hospital in July 2025 due to lumbar pain without obvious cause and a weight loss of 3 kg. Physical examination revealed multiple enlarged lymph nodes throughout the body, with the largest one located in the left cervical region, measuring approximately 7.0cm x3.0cm, firm in consistency, mobile, and non-tender. Splenomegaly was also noted, palpable below the costal margin.

Laboratory tests: White blood cell count 8.95×10^9^/L, lymphocyte count 1.13×10^9^/L, hemoglobin 120g/L, total platelet count 314×10^9^/L, absolute neutrophil count 7.26×10^9^/L; plasma EB virus nucleic acid test 1.46×10² copies/ml(reference range: not detected; lower limit of detection: 10 copies/mL), nucleated cell EB virus nucleic acid test 6.20×10² copies/ml, β2-microglobulin 4.39mg/L, lactate dehydrogenase 421U/L. The dynamic monitoring results of plasma EBV DNA load are shown in **Table 1**.

**Table 1:** Dynamic monitoring results of plasma EBV DNA load.

Time	EBV load (copies/mL)	Number of tests	Interpretation
2019-11-05 (at initial DLBCL diagnosis)	697.96	1	Elevated
2020–01 to 2020-08 (during treatment)	<10	4	Normal
2020–08 to 2025-07 (disease-free interval)	Not tested	0	Unknown
2025-07-31 (at CHL diagnosis)	146	1	Elevated
2025–09 to 2026-05 (during treatment)	<10	7	Normal

**Figure 2:**
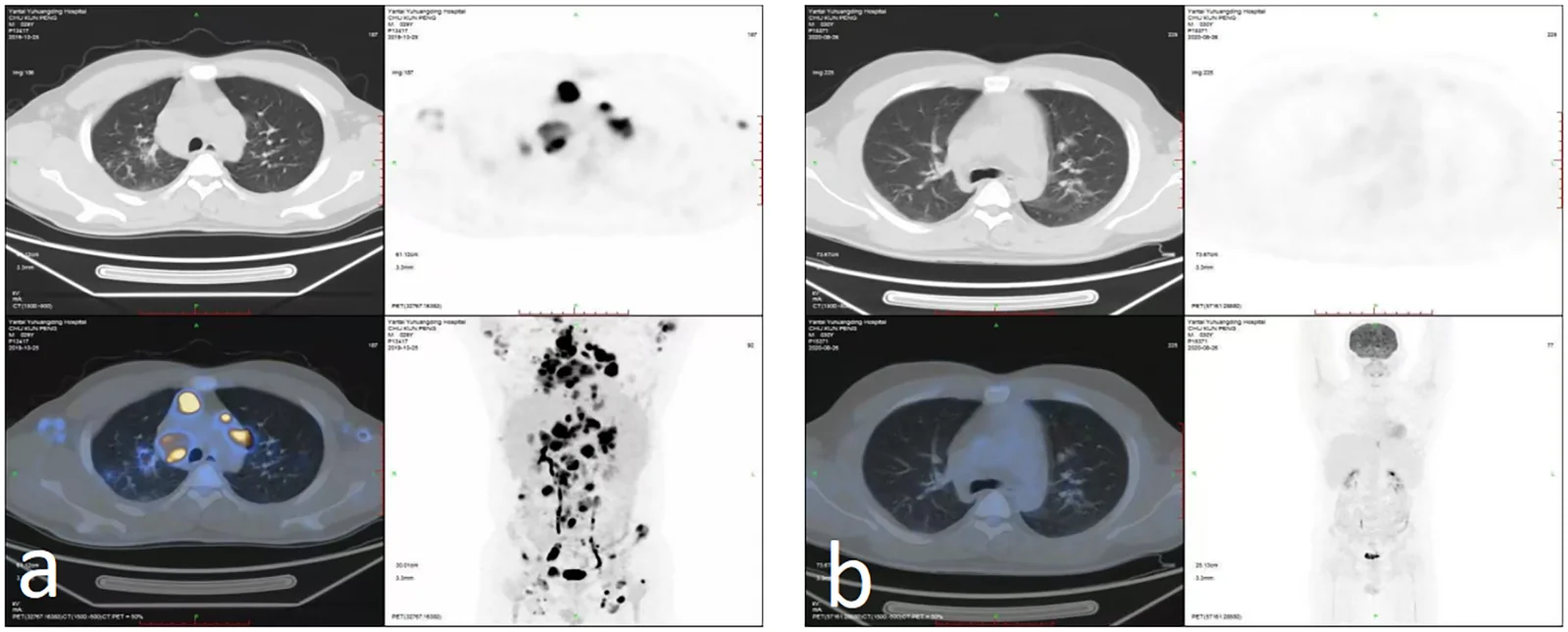
Imaging examinations before and after treatment. **(A)** Whole-body PET-CT shows uneven thickening of the bilateral and posterior walls of the nasopharynx; multiple lymph nodes throughout the body, including bilateral preauricular, right retroauricular, and bilateral cervical regions, exhibit intense FDG uptake; **(B)** PET-CT reveals no abnormal FDG metabolic lesions.

**Figure 3:**
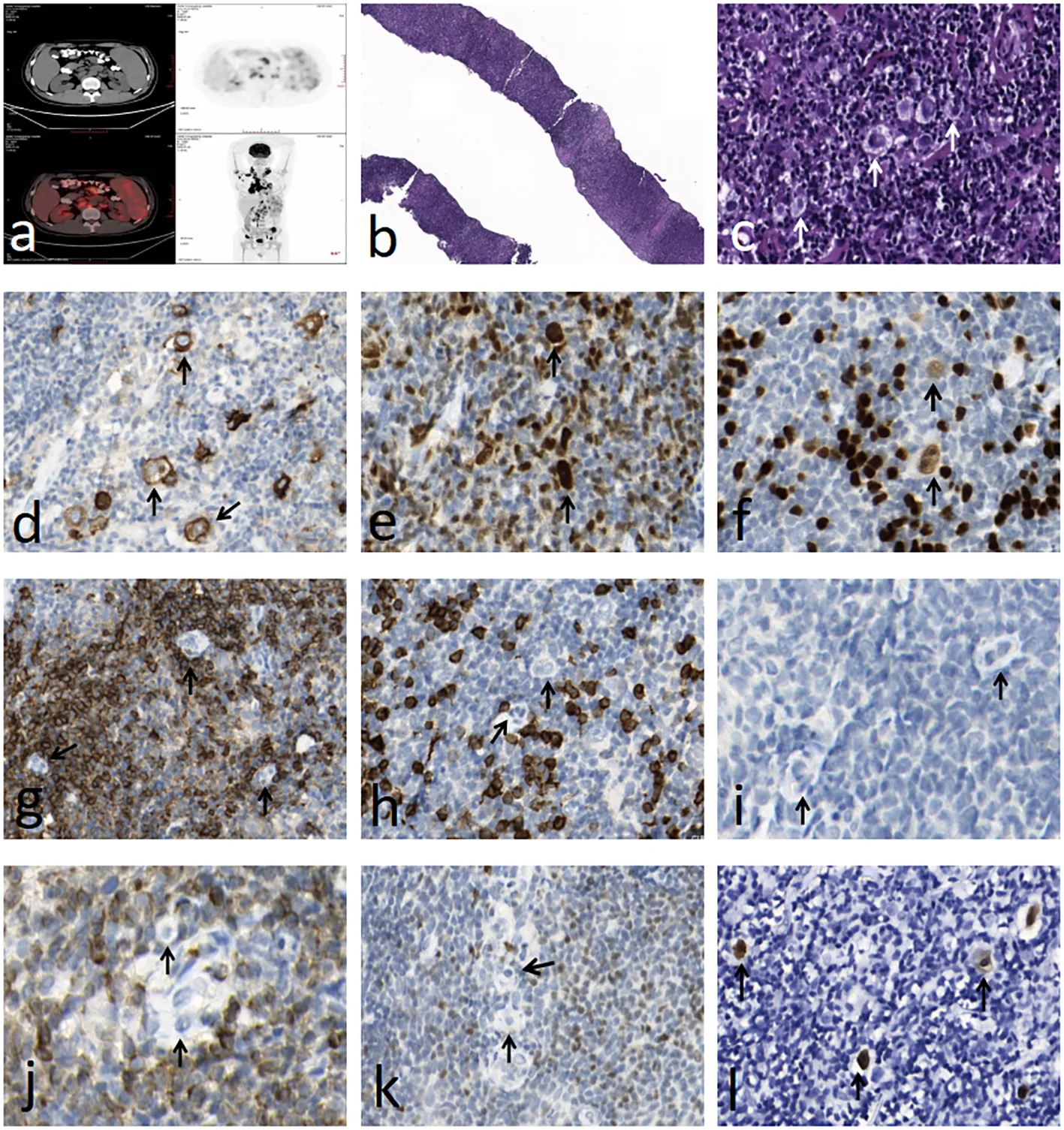
Pathological and imaging examinations of secondary tumors. **(A)** PET-CT reveals systemic multiple lymphadenopathy with increased FDG metabolism. **(B, C)** HE staining shows destruction of lymph node structure and lymphoid hyperplasia, predominantly composed of medium-to-large lymphocytes with irregular nuclei,scattered Reed-Sternberg cells (R-S cells) exhibiting large, binucleated or multinucleated nuclei with eosinophilic nucleoli, and a mixed inflammatory cell background. Immunohistochemical analysis of the left cervical lymph node demonstrates tumor cells (arrows) positive for CD30 **(D)**, MUM1 **(E)**, and PAX5 **(F)**, but negative for CD20 **(G)**, CD79a **(H)**, CD10 **(I)**, BCL-2 **(J)**, and BCL-6 **(K)**. **(L)** ISH analysis demonstrated positivity for Epstein-Barr virus-encoded small RNA in approximately 70% of Reed-Sternberg cells and variant large cell nuclei. Original magnification: **(B)** ×5.0; **(C–K)** ×90; **(L)** ×70.0. Background small lymphocytes are positive for CD20 and CD79a, while Reed-Sternberg cells are negative.

In October 2019, when the patient was first admitted to the hospital, PET-CT showed systemic multiple lymphadenopathy and increased bone metabolism, suggesting lymphoma with bone marrow infiltration (**Figure 2A**). During treatment, PET-CT was re-examined in February 2020, indicating partial remission. After treatment completion, PET-CT was re-examined in August 2020, showing complete metabolic response with a Deauville score of 1 (**Figure 2B**). In July 2025, the patient underwent PET-CT again due to symptom recurrence, showing systemic multiple lymphadenopathy and increased bone metabolism with a Deauville score of 5, suggestive of lymphoma recurrence (**Figure 3A**).

In August 2025, the patient underwent a needle biopsy of the left cervical lymph node. Gross examination revealed two tissue specimens, each measuring 0.8 cm in length and 0.1 cm in diameter. Microscopic examination showed destruction of the lymph node architecture with lymphoid tissue hyperplasia and infiltration by small lymphocytes, histiocytes, plasma cells, and eosinophils. Scattered Reed-Sternberg cells with abundant cytoplasm, coarse chromatin, large nuclei, binucleated or multinucleated forms, and prominent nucleoli were observed within the mixed inflammatory background (**Figures 3B, C**). Immunohistochemistry showed large cell markers: CD30 positive (**Figure 3D**), MUM1 positive (**Figure 3E**), PAX5 weakly positive (**Figure 3F**), C-myc positive (approximately 30%), p53 positive (approximately 40%), Ki-67 positive (approximately 90%). CD21 demonstrated a positive FDC mesh. CD3, CD20 (**Figure 3G**), CD79a (**Figure 3H**), CD10 (**Figure 3I**), BCL-2 (**Figure 3J**), BCL-6 (**Figure 3K**), CD5, cyclinD1, and CD19 were negative. EBV-EBER *in situ* hybridization (ISH) showed positivity in approximately 70% of the nuclei of Reed–Sternberg cells and variant large cells (**Figure 3L**). In conclusion, the patient was diagnosed with EBV-positive classical Hodgkin lymphoma (mixed cellularity type), Ann Arbor stage IV (by PET-CT; bone marrow biopsy not performed), IPS score 2 (low-intermediate risk).

The patient then continued to complete another 2 cycles of ABVD chemotherapy. In December 2025, a follow-up PET-CT scan revealed increased metabolism in the nasopharynx, palate, base of tongue, and bilateral tonsils, as well as multiple enlarged lymph nodes in the bilateral neck, with a Deauville score of 5, indicating lymphoma progression. To confirm the diagnosis, the patient underwent another needle biopsy of the supraclavicular lymph node, obtaining two tissue specimens measuring 1.5–2 cm in length and 0.1 cm in diameter. Immunohistochemistry showed CD30 positivity, CD45 negativity, PAX5 weak positivity, CD20 negativity, CD79a negativity, and EBV-EBER positivity in approximately 70% of cells. The pathological findings were completely consistent with the first biopsy, and the diagnosis was EBV-positive CHL (mixed cellularity type). After the diagnosis was confirmed, the patient started treatment with the BV+GemOX regimen (BV: brentuximab vedotin; GemOX: gemcitabine in combination with oxaliplatin. As of the last follow-up, 5 cycles had been completed, and the patient was still under treatment. We will continue to follow up with the patient.

## 3 Discussion

Composite Lymphoma (CL) refers to the simultaneous or sequential detection of two or more distinct types of lymphoma in the same patient, occurring at either identical or different anatomical sites (2). CL is clinically rare, with an annual incidence accounting for only 1%-4.7% of all new lymphoma cases (1). No single definitive mechanism has been proposed to explain the pathogenesis of different types of CL, as the etiology is considered diverse and complex, varying according to lymphoma types (5). These uncertainties pose certain challenges for diagnosis and treatment. Without sufficient DNA for clonality testing, the following mechanistic discussion is speculative and lacks molecular confirmation.

In this case, the patient presented with a nasal cavity lesion and EBV positivity at initial diagnosis, which required differentiation from extranodal NK/T-cell lymphoma (nasal type). The latter typically expresses CD56 and cytotoxic molecules, is negative for B-cell markers, and is often accompanied by angiodestruction and necrosis. In this case, the tumor cells were strongly positive for CD20 and CD79a, negative for CD3, and showed no angiodestruction or necrosis, consistent with EBV-positive DLBCL. Although CD56 and cytotoxic markers were not tested, the morphology and B-cell immunophenotype were not typical of NK/T-cell lymphoma. Subsequently, the patient received 1 cycle of CHOP regimen followed by 7 cycles of DA-EPOCH-R regimen. After 69 months, a needle biopsy was performed at the same site, with pathological diagnosis revealing EBV-positive CHL. Microscopically: large nuclei, binucleated or multinucleated Reed-Sternberg cells with eosinophilic nucleoli were scattered among small lymphocytes, histiocytes, plasma cells, and eosinophils, showing CD30 positivity and EBER expression. While DLBCL recurrence often expresses B-cell markers such as CD20 and CD79a, the Reed-Sternberg cells of CHL typically express CD30, PAX-5, and MUM1 but lack CD20 and LCA expression. The pathological features in this case were classic, confirming the diagnosis of metachronous EBV-positive CHL. Notably, both the initial DLBCL and the subsequent CHL in this case were CD30 positive. CD30 positivity is relatively rare in DLBCL but is a typical feature of CHL. CD30 positivity is a typical feature of CHL, but it can also be expressed in DLBCL. The expression of CD30 in both lymphoma components suggests possible lineage plasticity or a common differentiation pathway. EBV infection has been demonstrated to upregulate CD30 expression via the LMP1-NF-κB signaling pathway and may be involved in this process (6). The anti-CD30 antibody-drug conjugate brentuximab vedotin has potential therapeutic value in such CD30-positive EBV-associated lymphomas, but further studies are needed to validate its efficacy. Currently, there is no unified standard for the EBER positivity threshold: the ICC 2022 classification requires positivity in over 80% of lesional cells, whereas the WHO only requires EBV positivity in the majority of tumor cells (7). In this case, the 5th edition of the WHO classification of haematolymphoid neoplasms was adopted as the threshold for determining EBV positivity. At the same puncture site, the majority of tumor cells showed EBER-positive expression. Moreover, the plasma EBV load was significantly higher than normal levels at the time of both episodes, while it decreased to within the normal range during the treatment periods. These findings support a possible role for EBV in the pathogenesis of the lymphomas in this patient.

Epstein-Barr virus (EBV) is a gammaherpesvirus with an infection rate exceeding 95% in the human population (8). Previous studies have confirmed that EBV infection plays a significant role in the development and progression of various lymphomas, including Burkitt lymphoma, classical Hodgkin lymphoma, post-transplant lymphoproliferative disorder, extranodal NK/T-cell lymphoma, and diffuse large B-cell lymphoma (9). Composite lymphomas consisting of classical Hodgkin lymphoma and non-Hodgkin lymphoma are particularly rare, with an incidence significantly lower than other combination forms (3). To systematically retrieve literature on EBV-positive DLBCL combined with CHL as composite lymphoma, we searched four databases in December 2025: PubMed, Embase, Cochrane Library, and Web of Science Core Collection. The search terms were as follows: epstein-barr virus OR EBV AND diffuse large B-cell lymphoma OR DLBCL AND classical Hodgkin lymphoma OR CHL OR Hodgkin AND composite lymphoma OR sequential lymphoma. The search time range was from database inception to December 2025. Inclusion criteria were: pathologically confirmed cases of EBV-positive DLBCL and EBV-positive CHL occurring in the same patient, including synchronous and metachronous onset. Exclusion criteria were: cases where EBV status was not clearly reported or one of the lymphomas was EBV-negative, as well as studies published only as abstracts without full text availability. Literature screening was independently performed by two authors. Ultimately, we identified only 7 reported cases of CL that met the criteria. The detailed clinical data of all published relevant cases are summarized in **Table 2** (4, 10–15).

**Table 2:** Clinical data of previously published cases of EBV-positive DLBCL associated with CHL.

No.	Age/ Gender	Background pathogenic status	Co-occurrence status	Time interval (mo)	Histology sequence	Anatomical site	CHL subtype	EBV	Clonality association	Initial treatment regimen	Status	Reference
1	35/M	Hepatitis B, post-chemotherapy immunosuppression	Metachronous	69	DLBCL→CHL	Nasal cavity, LN	MC	+/+	–	CHOP×1;DA-EPOCH-R×7	Alive, under treatment	Present case
2	72/M	Advanced age, post-chemotherapy immunosuppression	Metachronous	6	CHL→DLBCL	Rectum→Lung	MC	+/+	–	Brentuximab + AVD ×6	Alive, under treatment	(10)
3	54/M	No immunodeficiency	Synchronous	—	FL → DLBCL/CHL^†^	LN	–	−/+/+	Clonally identical	R-CHOP ×6	ANED	(11)
4	38/M	No immunodeficiency	Metachronous + Synchronous	2	CHL→EBVMCU + DLBCL	LN→Colorecta, Liver	LD	+/+/+	Clonally distinct	ABVD ×2; BEACOPP ×4	NA	(4)
5	58/M	No immunodeficiency	Synchronous	—	CHL + DLBCL	LN, bone marrow	NS	+/+	–	R-ABVD ×3	DOD	(12)
6	44/M	Hepatitis B, AIDS	Synchronous	—	CHL + DLBCL	LN, liver, spleen	MC	+/+	–	ProMACE/MOPP	DOD	(13)
7	6/F	Primary immunodeficiency	Metachronous	24, 6	DLBCL→DLBCL→CHL	LN	LD	+/+/+	Clonally distinct	R-CHOP ×9	DOD	(14)
8	71/M	Advanced age, post-chemotherapy immunosuppression	Metachronous	12, 36	CHL→DLBCL→CHL	LN	MC	+/+	–	ABVD ×6	DOD	(15)

DLBCL, diffuse large B-cell lymphoma; CHL, classical Hodgkin lymphoma; EBV, Epstein–Barr virus; FL, follicular lymphoma; EBVMCU, EBV-positive mucocutaneous ulcer; LN, lymph node; MC, mixed cellularity; NS, nodular sclerosis; LD, lymphocyte depletion; ANED, alive with no evidence of disease; DOD, died of disease; NA, not available, patient lost to follow-up; ND, not done. †In that case, the DLBCL component arose from transformation of follicular lymphoma, which is pathogenetically different from the primary EBV-positive DLBCL in the present case. Readers should be aware of this difference when making comparisons.

Notably, the case reported by Karube et al. included an EBV-positive mucocutaneous ulcer (EBVMCU) component. According to the 5th edition of the WHO classification of haematolymphoid neoplasms, EBVMCU is an independent low-grade EBV-associated lymphoproliferative disorder, typically presenting as a localized ulcer of the skin or mucosa with a good prognosis, and is significantly different from EBV-positive DLBCL and CHL in terms of clinical presentation and biological behavior (16). Although this case contained the unique EBVMCU component, its CHL and DLBCL components were both EBV-positive, meeting the inclusion criteria of this study.

Previous studies have shown that over 70% of patients with composite lymphoma are aged ≥55 years, suggesting that aging may be a potential risk factor for the development of CL (17). However, as shown in **Table 2**, among the 8 cases of EBV-positive CL, 7 were male and 1 was female, with an age distribution ranging from 6 to 72 years. This does not align with the typical elderly population characteristics of CL, and may be related to the clear immunodeficiency background observed in these patients, including advanced age, post-chemotherapy immunosuppression, hepatitis B, AIDS, or primary immunodeficiency. Regarding clinical features, lymph node involvement was present in 7 out of the 8 cases. The onset type was predominantly metachronous, with synchronous cases being relatively rare. Pathologically, CHL mainly presented as the mixed cellularity subtype, and tumor cells in all cases were EBER-positive, strongly suggesting that EBV infection plays a key driving role in the development of this type of composite lymphoma.

Unfortunately, the main limitation of this case report is the failure to perform molecular clonality analysis between the initial DLBCL and secondary CHL components. The paraffin-embedded tissue at initial diagnosis was exhausted after completing routine pathological diagnosis, immunohistochemistry, and EBER *in situ* hybridization, and thus sufficient high-quality DNA could not be extracted for clonality testing. Therefore, the following discussion regarding the clonal origin of the two lymphomas is hypothetical, based only on indirect evidence and literature review, without molecular verification from this case. The first possibility is that the two lymphomas represent independent clones. After primary infection, EBV establishes latency in memory B cells (18) and may be reactivated under certain conditions, inducing different B-cell clones to undergo independent malignant transformation (19). EBV can mimic B-cell receptor signaling through LMP2A, thereby rescuing germinal center B cells from apoptosis (20), while LMP1 persistently activates oncogenic pathways such as NF-κB and JAK/STAT, driving the transformation of different clones and ultimately giving rise to lymphomas with distinct morphologies, phenotypes, and clonal origins (21, 22). Karube et al. reviewed 9 cases of EBV-positive CHL with B-NHL and found that all evaluable cases demonstrated different clonal origins (4); humanized mouse models have also confirmed that EBV can simultaneously induce CHL-like and DLBCL-like lymphomas (23).This mechanism is consistent with the immunophenotypic and clonal analysis findings in most EBV-associated CL cases and represents the most widely recognized pathogenic pattern to date.

The second possibility is that the two lymphomas share a common clonal origin. Studies have confirmed that in some cases of composite lymphomas composed of CHL and NHL, the two components are clonally related (2), possibly arising from a common progenitor cell that diverges into different subtypes through distinct transformation events. EBV-positive lymphomas generally harbor fewer genomic alterations and rely more heavily on EBV latent gene expression and immune evasion (24–26), conferring greater phenotypic plasticity that predisposes them to histological and immunophenotypic transformation under pressures from the tumor microenvironment, therapy, or immune imbalance. Wang et al. reported a case of composite lymphoma with coexisting FL, EBV+ DLBCL, and EBV+ CHL, in which shared clonal origin was confirmed by immunoglobulin heavy chain gene rearrangement (11); Richter syndrome also suggests a similar clonal relationship before and after transformation to EBV-positive gray zone lymphoma (27). In the present case, the two lymphomas occurred 69 months apart, but neither hypothesis can be supported due to the lack of molecular clonality analysis. Although the long interval may favor independent clonal origins, this remains speculative and requires molecular confirmation.

In EBV-positive DLBCL, the mutation frequency of the B2M gene is significantly increased, and loss of its function can lead to downregulation of MHC-I molecules and immune escape, thereby promoting tumor development and progression (28). Elevated serum B2M level usually reflects lymphoma tumor burden and disease activity, but this marker cannot be directly equated with B2M gene mutation. In this case, the patient showed persistently elevated serum B2M levels on multiple tests, suggesting high disease activity. However, since B2M mutation analysis was not performed, the presence of a loss-of-function B2M mutation could not be inferred. Nevertheless, for patients with EBV-positive DLBCL, persistently elevated serum B2M levels still suggest the need for clinical vigilance regarding high tumor burden and possible disease progression, including the risk of developing a secondary lymphoma. In summary, most patients with malignant lymphoma present with immune dysregulation and immune escape mechanisms (29). Both DLBCL and CHL are EBV-associated lymphomas with complex microenvironments, and this abnormal microenvironment creates favorable conditions for the development of secondary lymphomas.

The influence of treatment-related factors should not be overlooked either. The standard treatment for malignant lymphoma involves systemic chemotherapy combined with radiotherapy. Composite lymphoma (CL) can be classified into synchronous or metachronous onset. For synchronous CL, the treatment strategy should primarily be based on the more aggressive subtype; for metachronous CL, individualized diagnosis and treatment should be implemented according to different pathological types. This case is metachronous CL. The patient had a history of hepatitis B but no other known immunodeficiency. The immunosuppressive state following multi-cycle chemotherapy may have also contributed to the development of the secondary lymphoma. In this case, due to high tumor burden, the patient received CHOP for initial debulking followed by a more intensive DA-EPOCH-R regimen, which contained rituximab, etoposide, and continuous infusion. Although the patient achieved complete remission, his immune function was suppressed after treatment, further reducing the ability to clear EBV-infected cells and creating conditions for CL development. In addition, the accumulation of chemotherapeutic drug doses was also an important contributing factor (30). Studies have shown that patients receiving more than 8 cycles of CHOP chemotherapy have a higher probability of developing secondary tumors, among which hematologic malignancies accounted for approximately 35.2% (31). Among the 8 cases of EBV-positive CL, 5 were metachronous onset, and all these patients received at least 6 cycles of chemotherapy (4, 10, 11, 14, 15). This demonstrates that regardless of the baseline immune status, prolonged multi-cycle chemotherapy-induced immunosuppression and drug accumulation may create favorable conditions for EBV-driven secondary lymphoma.

With the widespread adoption of monoclonal antibodies, the first-line treatment for DLBCL patients has evolved from traditional chemotherapy to rituximab combined with chemotherapy, leading to a significant improvement in the five-year survival rate (32, 33). However, DLBCL patients who received rituximab treatment and survived for 5 years have an incidence of secondary malignancies approximately 1.5 times higher than that of the general population (34), further emphasizing the importance of long-term regular follow-up and tumor surveillance.

Patients with EBV-positive status generally have poorer overall survival outcomes (29). Among the 8 cases of EBV-positive CL summarized in this study, 4 ultimately succumbed to the disease, 1 achieved disease-free survival, 1 was lost to follow-up, and 2 patients were still under treatment. Available reported cases suggest that outcomes may be poor, although conclusions are limited by the small number of cases and heterogeneous clinical settings. In this case, PD-L1 expression was not tested. However, given that PD-L1 overexpression in EBV-positive lymphomas has been reported in the literature (35). Therefore, for patients with EBV-positive CL, the value of PD-L1 expression testing and immune checkpoint inhibitor therapy can be further explored in the future, although this requires more research to validate.

In summary, composite lymphoma with EBV-positive DLBCL and CHL is extremely rare, exhibiting complex clinical manifestations and pathological features that are prone to misdiagnosis or missed diagnosis. This case report and literature review suggest that most such patients have an immunodeficient background, with both lymphoma components closely associated with EBV infection. The immunosuppressed state following prolonged, multi-cycle chemotherapy may create conditions for EBV-driven malignant transformation of independent B-cell clones. It is worth noting that this patient was lost to follow-up after achieving complete remission, resulting in a 5-year gap in surveillance, and did not seek medical attention again until clinical symptoms appeared, when CHL was diagnosed. This highlights that for patients with EBV-positive DLBCL, even after complete remission, long-term regular follow-up and dynamic monitoring of EBV viral load are essential. Additionally, early detection of PD-L1 expression in tumor tissue and timely initiation of immune-targeted therapy may improve the prognosis of such patients. The main limitation of this case report is the lack of molecular clonality analysis data. Therefore, the above mechanistic discussion regarding clonal origin is only hypothetical. In the future, more cases need to be accumulated and molecular clonality analysis and microenvironment studies should be conducted to further elucidate the pathogenesis and optimize diagnostic and therapeutic strategies. Furthermore, excisional biopsy is the gold standard for diagnosing CHL. However, in this case, core needle biopsy was chosen because the recurrent lymph node was located deep in the neck, adjacent to important blood vessels, and the risk of excision was high. Although the second biopsy obtained more abundant tissue and yielded consistent results, the inability to obtain the complete lymph node structure remains a limitation of this case.

## Data Availability

The raw clinical and pathological datasets used in this study are confidential and restricted to internal hospital access only. They are not publicly available due to patient privacy protection and hospital data management regulations. All restricted clinical data are stored in the local hospital database. Access is limited to authorized researchers only. Qualified researchers may contact the corresponding author to submit a formal data access application with ethical approval.

## References

[1] Thirumala S Esposito M Fuchs A . An unusual variant of composite lymphoma: a short case report and review of the literature. Arch Pathol Lab Med. (2000) 124:1376–8. doi: 10.1016/S1470-2045(14)70153-6 10975943

[2] Küppers R Dührsen U Hansmann ML . Pathogenesis, diagnosis, and treatment of composite lymphomas. Lancet Oncol. (2014) 15:e435–446. doi: 10.5858/2000-124-1376-AUVOCL 25186047

[3] Yu G Kong L Qu G Zhang Q Wang W Jiang L . Composite lymphoma in the anterior mediastinum: a case report and review of the literature. Diagn Pathol. (2011) 6:60. doi: 10.1186/1746-1596-6-60 21733146 PMC3141624

[4] Karube K Takatori M Kohno K Tomoyose T Ohshiro K Nakazato I . Co-occurrence of EBV-positive classic hodgkin lymphoma and B-cell lymphomas of different clonal origins: a case report and literature review. Pathol Int. (2020) 70:893–8. doi: 10.1111/pin.13012 32881147

[5] Mokhtar NM . Review article composite lymphoma. J Egyptian Natl Cancer Institute. (2007) 19:171–5. 19190689

[6] Wang WT Xing TY Du KX Hua W Guo JR Duan ZW . CD30 protects EBV-positive diffuse large B-cell lymphoma cells against mitochondrial dysfunction through BNIP3-mediated mitophagy. Cancer Lett. (2024) 583:216616. doi: 10.1016/j.canlet.2024.216616 38211650

[7] Kurz KS Ott M Kalmbach S Steinlein S Kalla C Horn H . Large B-cell lymphomas in the 5th edition of the WHO-classification of haematolymphoid neoplasms-updated classification and new concepts. Cancers. (2023) 15:2285. doi: 10.3390/cancers15082285 37190213 PMC10137297

[8] Cohen JI Jaffe ES Dale JK Pittaluga S Heslop HE Rooney CM . Characterization and treatment of chronic active Epstein-Barr virus disease: a 28-year experience in the United States. Blood. (2011) 117:5835–49. doi: 10.1182/blood-2010-11-316745 21454450 PMC3112034

[9] Zhou J Xiao Y . research progress on EB virus and its related lymphoma - review. Zhongguo Shi Yan Xue Ye Xue Za Zhi. (2018) 26:292–5. doi: 10.7534/j.issn.1009-2137.2018.01.052 29397861

[10] Di J Wei S Jackson A Munker R Kesler MV . Transition from epstein-barr virus (EBV)-positive rectal hodgkin lymphoma to diffuse large B-cell lymphoma in the lung. Cureus. (2024) 16:e65013. doi: 10.7759/cureus.65013 39165470 PMC11333787

[11] Ryder CB Saeed H Hussaini M . Composite lymphoma with follicular lymphoma transformation to clonally related epstein-barr virus (EBV) positive diffuse large B-cell lymphoma and EBV-PositiveClassic hodgkin lymphoma. Case Rep Hematol. (2023) 2023:8833273. doi: 10.1155/2023/8833273 38028985 PMC10651334

[12] Hwang YY Leung AYH Lau WH Loong F So JCC Tse E . Synchronous epstein–barr virus-positive diffuse large B-cell lymphoma of the elderly and epstein–barr virus-positive classical hodgkin lymphoma. Histopathology. (2011) 59:352–5. doi: 10.1111/j.1365-2559.2011.03905.x 21884219

[13] Guarner J Rio CD Hendrix L Unger ER . Composite hodgkin’s and non-hodgkin’s lymphoma in a patient with acquired immune deficiency syndrome. In-situ demonstration of epstein–barr virus. Cancer. (1990) 66:796–800. doi: 10.1002/1097-0142(19900815)66:4<796::aid-cncr2820660433>3.0.co;2-u 2167145

[14] Bautista-Quach MA Bedros A Wang J . Subsequent development of diffuse large B-cell lymphomas and hodgkin lymphoma associated with primary immune disorder in a 6-year-old female: a case report and review of the literature. J Pediatr Hematology/Oncology. (2011) 33:e320–5. doi: 10.1097/mph.0b013e318207a37a 21572347

[15] Murase T Fujita A Ueno H Park JW Yano T Hoshikawa M . A case of age-related EBV-associated B-cell lymphoproliferative disorder metachronously showing two distinct morphologic appearances, one of a polymorphic disease resembling classical hodgkin lymphoma, and the other of a large-cell lymphoma. Int J Hematol. (2009) 89:80–5. doi: 10.1007/s12185-008-0214-0 19093168

[16] Ikeda T Gion Y Nishimura Y Nishimura MF Yoshino T Sato Y . Epstein–barr virus-positive mucocutaneous ulcer: a unique and curious disease entity. Int J Mol Sci. (2021) 22:1053. doi: 10.3390/ijms22031053 33494358 PMC7865427

[17] Giua R Fontana D Deda G Bianchi A Rabitti C Antonelli Incalzi R . Composite mantle-cell lymphoma and classical Hodgkin lymphoma in a very old adult. J Am Geriatrics Soc. (2015) 63:824–6. doi: 10.1111/jgs.13355 25900501

[18] Carbone A Gloghini A Dotti G . EBV-associated lymphoproliferative disorders: classification and treatment. Oncologist. (2008) 13:577–85. doi: 10.1634/theoncologist.2008-0036 18515742

[19] Zettl A Lee SS Rüdiger T Starostik P Marino M Kirchner T . Epstein-Barr virus-associated B-cell lymphoproliferative disorders in angloimmunoblastic T-cell lymphoma and peripheral T-cell lymphoma, unspecified. Am J Clin Pathol. (2002) 117:368–79. doi: 10.1309/6utx-gvc0-12nd-jjeu 11888076

[20] Mancao C Altmann M Jungnickel B Hammerschmidt W . Rescue of “crippled” germinal center B cells from apoptosis by epstein-barr virus. Blood. (2005) 106:4339–44. doi: 10.1182/blood-2005-06-2341 16076866 PMC1895254

[21] Vaysberg M Lambert SL Krams SM Martinez OM . Activation of the JAK/STAT pathway in epstein barr virus+-associated posttransplant lymphoproliferative disease: role of interferon-gamma. Am J Transplantation: Off J Am Soc Transplant Am Soc Transplant Surgeons. (2009) 9:2292–302. doi: 10.1111/j.1600-6143.2009.02781.x 19656130 PMC2774223

[22] Kato H Karube K Yamamoto K Takizawa J Tsuzuki S Yatabe Y . Gene expression profiling of epstein-barr virus-positive diffuse large B-cell lymphoma of the elderly reveals alterations of characteristic oncogenetic pathways. Cancer Sci. (2014) 105:537–44. doi: 10.1111/cas.12389 24581222 PMC4317839

[23] Li C Romero-Masters JC Huebner S Ohashi M Hayes M Bristol JA . EBNA2-deleted epstein-barr virus (EBV) isolate, P3HR1, causes hodgkin-like lymphomas and diffuse large B cell lymphomas with type II and wp-restricted latency types in humanized mice. PloS Pathog. (2020) 16:e1008590. doi: 10.1371/journal.ppat.1008590 32542010 PMC7316346

[24] Yoon H Park S Ju H Ha SY Sohn I Jo J . Integrated copy number and gene expression profiling analysis of epstein-barr virus-positive diffuse large B-cell lymphoma. Genes Chromosomes Cancer. (2015) 54:383–96. doi: 10.1002/gcc.22249 25832818

[25] Vermaat JS Somers SF de Wreede LC Kraan W de Groen RAL Schrader AMR . MYD88 mutations identify a molecular subgroup of diffuse large B-cell lymphoma with an unfavorable prognosis. Haematologica. (2020) 105:424–34. doi: 10.3324/haematol.2018.214122 31123031 PMC7012469

[26] Kong IY Giulino-Roth L . Targeting latent viral infection in EBV-associated lymphomas. Frontiers in Immunology. (2024) 15:1342455. doi: 10.3389/fimmu.2024.1342455 38464537 PMC10920267

[27] Liu Y Ho C Roshal M Baik J Arcila M Zhang Y . Transformation of monoclonal B lymphocytosis to epstein-barr virus-positive large B-cell lymphoma with intermediate features between diffuse large B-cell lymphoma and classic hodgkin lymphoma. AJSP: Rev Rep. (2019) 24:207–11. doi: 10.1097/pcr.0000000000000326 33437870 PMC7799843

[28] He M Liu B Tang G Jiao L Liu X Yin S . B2M mutation paves the way for immune tolerance in pathogenesis of epstein-barr virus positive diffuse large B-cell lymphomas. J Cancer. (2022) 13:3615–22. doi: 10.7150/jca.75813 36606194 PMC9809314

[29] Song CG Huang JJ Li YJ Xia Y Wang Y Bi XW . Epstein-barr virus-positive diffuse large B-cell lymphoma in the elderly: A matched case-control analysis. PloS One. (2015) 10:e0133973. doi: 10.1371/journal.pone.0133973 26222726 PMC4519250

[30] Xu Y Wang H Zhou S Yu M Wang X Fu K . Risk of second Malignant neoplasms after cyclophosphamide-based chemotherapy with or without radiotherapy for non-hodgkin lymphoma. Leukemia Lymphoma. (2013) 54:1396–404. doi: 10.3109/10428194.2012.743657 23101661

[31] Xu YL Wang HQ Qian ZZ Zhou SY Zhang HL Qiu LH . Clinical Analysis of Non-Hodgkin's Lymphoma Associated with Secondary Malignant Neoplasm. Chin J Clin Oncol. (2012) 39:1426–29, 1433. doi: 10.3969/j.issn.1000-8179.2012.19.009

[32] Pfreundschuh M Kuhnt E Trümper L Osterborg A Trneny M Shepherd L . CHOP-like chemotherapy with or without rituximab in young patients with good-prognosis diffuse large-B-cell lymphoma: 6-year results of an open-label randomised study of the MabThera international trial (MInT) group. Lancet Oncol. (2011) 12:1013–22. doi: 10.1016/s1470-2045(11)70235-2 21940214

[33] Coiffier B Lepage E Briere J Herbrecht R Tilly H Bouabdallah R . CHOP chemotherapy plus rituximab compared with CHOP alone in elderly patients with diffuse large-B-cell lymphoma. N Engl J Med. (2002) 346:235–42. doi: 10.1056/nejmoa011795 11807147

[34] Geurts YM Neppelenbroek SIM Aleman BMP Janus CPM Krol ADG van Spronsen DJ . Treatment-specific risk of subsequent Malignant neoplasms in five-year survivors of diffuse large B-cell lymphoma. ESMO Open. (2024) 9:102248. doi: 10.1016/j.esmoop.2024.102248 38350338 PMC10937196

[35] Xie W Medeiros LJ Li S Tang G Fan G Xu J . PD-1/PD-L1 pathway: a therapeutic target in CD30+ large cell lymphomas. Biomedicines. (2022) 10:1587. doi: 10.3390/biomedicines10071587 35884893 PMC9313053

